# Postpartum Methicillin-Resistant* Staphylococcus aureus* Toxic Shock Syndrome Caused by a Perineal Infection

**DOI:** 10.1155/2018/2670179

**Published:** 2018-09-30

**Authors:** Yoko Deguchi, Yuko Horiuchi, Kensaku Shojima, Naoyuki Iwahashi, Miwa Ikejima, Kazuhiko Ino, Kenichi Furukawa

**Affiliations:** ^1^Department of Obstetrics and Gynecology, Hashimoto Municipal Hospital, Wakayama, Japan; ^2^Department of General Internal Medicine, Hashimoto Municipal Hospital, Wakayama, Japan; ^3^Department of Obstetrics and Gynecology, Wakayama Medical University, Wakayama, Japan

## Abstract

Although toxic shock syndrome (TSS) is rare, multiorgan failure can occur without early identification and appropriate therapy. In particular, a few cases of postpartum TSS due to methicillin-resistant* Staphylococcus aureus* (MRSA) have been reported. Here, we describe a rare case in which a 32-year-old Japanese woman had TSS due to MRSA that was caused by a perineal infection after a normal vaginal delivery. Twelve days after giving birth to a healthy child, she was readmitted to our hospital due to a 2-day fever and perineal pain without uterine tenderness. She developed emesis and watery diarrhea on the night of admission. On the second day, a diffuse cutaneous macular rash appeared over her trunk. Laboratory data revealed deteriorated renal function and thrombocytopenia. Her history and clinical results were compatible with a typical course of TSS. Administration of ceftriaxone and clindamycin was started immediately after admission and was effective. The patient recuperated steadily over the next week with desquamation of the skin. MRSA was isolated from her vaginal discharge and was found to produce TSS toxin 1 (TSST-1). Furthermore, since MRSA was not detected in the nasal and vaginal cavity during pregnancy, it suggests that vaginal colonization can also occur postpartum and be the disease source in mothers. Therefore, MRSA infections should be considered when treating for postpartum TSS.

## 1. Introduction

Toxic shock syndrome (TSS) is characterized by high fever, erythematous rash with subsequent desquamation of the skin, shock, and multiple organ involvement and was first reported by Todd et al. in 1978 [1]. Although TSS has occurred predominantly in menstruating women, the proportion of cases not associated with menstruation has been increasing steadily since 1980 [2]. The removal of high-absorbency tampons from the market has dramatically decreased the incidence of menstrual TSS. As a result, approximately one-half of all reported TSS cases are currently nonmenstrual. Nonmenstrual TSS can occur due to the use of barrier contraceptives, vaginal and cesarean deliveries, surgical and postpartum wound infections, mastitis, septorhinoplasty (particularly when nasal packing is used), sinusitis, arthritis, burns, cutaneous and subcutaneous lesions (including abscesses, ulcers, and cellulitis), respiratory infections after influenza, and enterocolitis. In most cases of TSS associated with a surgical site or wound infections, it can occur in the absence of any signs of apparent infection [3].

Although TSS is known to be associated with staphylococcal infections in almost any sites in various settings, reports regarding TSS caused by methicillin-resistant* Staphylococcus aureus* (MRSA) are rare. A MEDLINE search from 1966 to 2017 revealed only two case reports (mastitis and endometritis; Table 1) of postpartum TSS caused by MRSA; however, there are no reports of postpartum MRSA-TSS caused by perineal lacerations [4, 5]. Here, we report a rare case of postpartum MRSA-TSS detected from a tiny perineal laceration.

## 2. Case Presentation

A 32-year-old Japanese woman (gravida 4, para 3) with no medical history was admitted to our hospital for a term delivery. She had a normal vaginal delivery of a healthy child at 39 weeks of gestation and underwent a suture of first-degree perineal laceration on the left side of the external urethral orifice. She used a perineal pad and no intravaginal packings after delivery. Furthermore, she did not have fever and wound pain during hospitalization. She was doing well and was discharged on the fifth day.

Twelve days after delivery, she presented with a 2-day history of fever of 40oC and focal perineal pain. On physical examination, the patient was conscious but had hypotension (88/56 mmHg), tachycardia (144 beats/minute), and tachypnea (23 breaths/minute). Her body temperature was 40.2°C. Abdominal examination findings were normal, and pelvic examination disclosed that the uterus and the vagina were not tender. Although the external genitalia around the laceration were accompanied by pain and fever, reddening and swelling were absent. Laboratory data revealed abnormal results; she had neutrophilic leukocytosis (white blood cells were 19,600/mm^3^ with 96.1% neutrophils, 1.2% lymphocytes and 2.4% monocytes), hypoproteinemia (6.6 g/dl), and hypoalbuminemia (3.6 g/dl). Furthermore, the serum electrolyte concentrations were abnormal: sodium, 136 mEq/L; potassium, 3.2 mEq/L; chloride, 97 mEq/L; calcium, 8.2 mg/dl; phosphate, 3.7 mg/dl; magnesium, 1.5 mg/dl.

She was treated with aggressive intravenous fluid resuscitation and antimicrobial agent therapy with intravenous ceftriaxone (2 g every 12 hours). On admission, vaginal, urine, stool, and blood cultures were performed prior to initiating the administration of antibiotics. She developed emesis and watery diarrhea on the night of admission. On the second hospitalization day, a diffuse cutaneous macular rash appeared over her trunk. Laboratory values indicated mild renal disturbance (BUN 19.5 mg/dL, creatinine 0.93 mg/dL, and eGFR 57.40), and she also had leukocytosis (WBC 13,400/*µ*L), thrombocytopenia (platelets 124,000/*µ*L), hyperbilirubinemia (total bilirubin 3.01 mg/dL), and elevated creatine kinase (319 IU/L). Since these symptoms and results were compatible with a typical course of TSS, clindamycin was also added to the antibiotic therapy. On hospitalization day 4, desquamation on her palms and fingers strongly suggested the diagnosis of TSS. Furthermore, the bacteriological results indicated that the TSS was due to methicillin-resistant* Staphylococcus aureus.* Although the urine, stool, and blood cultures were negative, cultures from the vagina yielded a significant growth of MRSA with dramatic reduction in genital flora that was resistant to ceftriaxone but susceptible to clindamycin. We routinely test the nares and vaginal cultures during pregnancy, but no bacterium including MRSA were not detected.

The treatment outcome was favorable. On hospitalization day 5, her vital signs became normal, the erythematous skin rash disappeared, and biological abnormalities were also resolved. She was discharged at the end of an 11-day course of antibiotic therapy (ceftriaxone for 7 days, clindamycin for 11 days). After discharge, the TSS toxin assay results were obtained. MRSA was isolated from the vaginal secretion and was found to produce TSS toxin-1 (TSST-1). One month after discharge, she has not had any recurrences, and the baby is doing well without infectious diseases.

## 3. Discussion

TSS is an acute life-threatening disease, and its early recognition is important in order to provide the appropriate therapy. Current diagnostic criteria for TSS are published by the Centers for Disease Control and Prevention (CDC). There are five clinical criteria for the diagnosis of TSS without assessing for streptococcal infections: fever over 38.9°C, rash, desquamation of the skin, hypotension, and the involvement of three or more organ systems including the gastrointestinal, muscular, mucous membranes, renal, hepatic, hematologic, and central nervous neurological systems. In addition, TSS can be confirmed by the following laboratory criteria: negative results for Rocky Mountain spotted fever, measles, or leptospirosis in blood, throat, and cerebrospinal cultures (blood cultures may be positive for* S. aureus*) [6]. In present case, a patient almost fulfilled the clinical criteria of TSS except for the desquamation of the skin on admission; she had a fever, rash, hypotension, and the involvement of at least three organ systems (vomiting, diarrhea, elevated creatine kinase, acute renal disturbance, and thrombocytopenia). Desquamation on her palms and fingers on hospitalization day 4 made the diagnosis of TSS definite.

In most cases of TSS associated with a surgical site or wound infections, there is little or no evidence of inflammation at the site of* S. aureus* replication. A careful examination is therefore required because delayed diagnosis of TSS is associated with increased morbidity and mortality [4]. In this patient, there was no evidence of preexisting intrauterine infection and no history of postpartum tampon use. Furthermore, she lacked clinical symptoms of intrauterine infections such as pain and tenderness in the uterus or abnormal lochia.

Postpartum MRSA-TSS is rare, and, to our knowledge, only 2 cases of postpartum MRSA-TSS have been reported in English literature (Table 1) [4, 5]. In our case, MRSA vaginal colonization or a tiny wound in the labium minora was thought to be the cause for TSS since there were few signs of infections from the pelvic examination except for the presence of pain. Although postpartum MRSA-TSS is rare, careful examinations should be performed since it can be caused by MRSA vaginal colonization or only a tiny wound.

*S. aureus*, including MRSA, often colonize the anterior nares of humans. Recent data from a national survey between 2001 and 2004 in the United States found that the prevalence of nasal MRSA colonization is increasing (from 0.8% to 1.5% of the population) despite an overall decrease in nasal* S. aureus* colonization. Risk factors for nasal MRSA colonization in the general population include the male gender, limited health care exposure, age 60 years and over, diabetes, and poverty [7]. Two recent prevalence studies in the United States reported rectovaginal MRSA colonization rates of 0.5–3.5% during pregnancy [8, 9] Only a fraction of those who have MRSA colonization are thought to go on to develop infections; however, MRSA was not detected in the nasal and vaginal cavity of our patient during pregnancy. The occurrence of the infection during her hospital stay after giving birth cannot be excluded. Therefore, we should consider the risk factors for MRSA infections in the health care setting. Transmission of toxin-producing MRSA in obstetrical wards among women and/or neonates has been described in cases of neonatal TSS-like exanthematous diseases [10–12]. CDC guidelines recommend the use of contact precautions, such as the use of gowns and gloves for direct contact with patients and their environment, for patients who are carriers of MRSA or have MRSA infections [13].

Early recognition of TSS is important in order to provide the appropriate therapy. This therapy should include the prompt drainage of the site to control ongoing toxin production, the use of antimicrobial therapy targeting* S. aureus* (such as clindamycin to inhibit protein synthesis and further toxin production, and vancomycin if MRSA is the causative organism), and supportive therapy including fluid resuscitation and pressor support, if required.

To our knowledge, this is the third report regarding postpartum MRSA-TSS. Gynecologists and obstetricians should be aware of the possibility of postpartum MRSA-TSS. The results from this case suggest that vaginal colonization can occur postpartum and be the source of disease in mothers. Furthermore, MRSA infections should be considered when treating postpartum TSS.

## Figures and Tables

**Table 1:** Definite cases of postpartum MRSA toxic shock syndrome.

First author	publication year	vaginal delivery or caesarean section	time TSS developed after delivery	complicating factor	cultures with MRSA	antibiotics	surgeries performed for control of infection	maternal outcome	detected toxin
J. I. Andrews [4]	2008	Caesarean section	4 weeks	mastitis	breast fluid	clindamycin piperacillin/ tazobactam vancomycin	none	survived	enterotoxin B
C. Collet [5]	2009	Vaginal delivery	4 weeks	endometritis	vaginal	Cefotaxime vancomycin metronidazole	hysterectomy	survived	TSST-1
present case	2017	Vaginal delivery	12 days	surgical wound	vaginal	Ceftriaxone clindamycin	none	survived	TSST-1
